# The autism-mutated ADNP plays a key role in stress response

**DOI:** 10.1038/s41398-019-0569-4

**Published:** 2019-09-18

**Authors:** Shlomo Sragovich, Yarden Ziv, Sharon Vaisvaser, Noam Shomron, Talma Hendler, Illana Gozes

**Affiliations:** 1 The Lily and Avraham Gildor Chair for the Investigation of Growth Factors, The Elton Laboratory for Neuroendocrinology, Department of Human Molecular Genetics and Biochemistry, Sackler Faculty of Medicine, Sagol School of Neuroscience and Adams Super Center for Brain Studies, Tel Aviv University, Tel Aviv, 69978 Israel; 2 Functional Brain Center, Wohl Institute for Advanced Imaging, Sourasky Medical Center, Tel Aviv, Israel; 3 Sackler Faculty of Medicine, Tel Aviv University, Tel Aviv, 69978 Israel; 4 School of Psychological Sciences, Sackler Faculty of Medicine, Sagol School of Neuroscience, Tel Aviv University, Tel Aviv, 69978 Israel

**Keywords:** Biomarkers, Neuroscience

## Abstract

Activity-dependent neuroprotective protein (ADNP), discovered and first characterized in our laboratory (IG), is vital for mammalian brain formation and presents one of the leading genes mutated de novo causing an autistic syndrome, namely the *ADNP* syndrome. Furthermore, a unique mouse model of *Adnp*-haploinsufficiency was developed in the laboratory (IG), with mice exhibiting cognitive and social deficiencies. ADNP is regulated by vasoactive intestinal peptide (VIP), and pituitary adenylate cyclase-activating peptide (PACAP). In this respect, PACAP was independently identified as a sexual divergent master regulator of the stress response. Here, we sought to determine the impact of the *Adnp* genotype and the efficacy of PACAP pre-treatment when subjecting *Adnp*^+/−^ mice to stressful conditions. Significant sex differences were observed with *Adnp*^+/−^ males being more susceptible to stress in the object and social recognition tests, and the females more susceptible in the open field and elevated plus maze tests. Splenic *Adnp* expression and plasma cortisol levels in mice were correlated with cognition (male mice) and anxiety-related behavior. These findings were further translated to humans, with observed correlations between *ADNP* expression and stress/cortisol content in a young men cohort. Altogether, our current results may establish ADNP as a marker of stress response.

## Introduction

Activity-dependent neuroprotective protein (ADNP), discovered and first characterized in our laboratory (IG) as vital for mammalian brain formation^1–5^, was found to be frequently mutated in autism spectrum disorder (ASD) with associated cognitive deficits^6^ (also known as the *ADNP* syndrome), as well as deficiencies in schizophrenia and Alzheimer’s disease (AD). In this respect, an 8-amino-acid peptide (NAPVSIPQ = NAP, also called davunetide or CP201) derived from ADNP, was likewise discovered in the Gozes laboratory, and shown to enhance cognitive function in *Adnp*^+/−^ mice^4,7,8^. NAP safety and efficacy profiles were further translated to humans, showing cognitive and functional protection in clinical trials involving patients suffering from amnestic mild cognitive impairment and schizophrenia^9–12^. ADNP is regulated by vasoactive intestinal peptide (VIP)^1^, as well as pituitary adenylate cyclase-activating peptide (PACAP)^13^, which change its content toward neuroprotection. Furthermore, PACAP has been previously identified as a sexual divergent master regulator of stress response, together with its receptor PAC1, and as such, these were tightly associated specifically with post-traumatic stress disorder (PTSD)^14–16^, in a sex-dependent manner^14,15,17^.

The *ADNP* syndrome is characterized by global developmental delays, intellectual disabilities (ID), speech impediments and motor dysfunctions^18–20^. While ADNP is vital for mammalian brain formation^1–3^, *Adnp*-haploinsufficient (*Adnp*^+/−^) mice survive^4^, presenting a preserved phenotype even when outbred^7,21,22^. This confirms the highly strong impact of the *Adnp* genotype, especially in terms of cognitive protection, thus predicting the human *ADNP* ID syndrome^4^. Furthermore, *Adnp*-haploinsufficient mice, expressing approximately half the content of Adnp (compared with *Adnp*^+/+^ mice)^7,21^, were recently shown to mimic the human *ADNP* syndrome patient in terms of delayed development, and motor impediments^7^. The mouse model allowed characterization at the memory synapse level, showing that *Adnp* deficiency reduced dendritic spine density and altered synaptic gene expression, both of which were partly ameliorated by treatment with the ADNP-derived snippet, drug candidate, NAP. Importantly, the global developmental delays, vocalization impediments, gait/motor dysfunctions and social/object memory impairments exhibited in *Adnp*^+/−^ mice, were all partially reversed by daily NAP administration (systemic/nasal)^7^. Thus, a better understanding of the *ADNP* syndrome was provided, paving the path for NAP (CP201) to clinical development in the *ADNP* syndrome children^7^.

An additional important aspect in developmental delays/ASD involves the element of stress and trauma^23^. ASD patients are known to suffer from difficulties encompassing social interaction and communication. These impediments may further lead to increased stress coupled with cognitive and emotional overload state^23^. Furthermore, the classification of events as traumatic (e.g. social insults and changes in known routines), may be defined by certain ASD characteristics, putting children diagnosed with ASD at increased risk for both encountering stressful events and developing subsequent posttraumatic sequelae^24–26^. In this respect, on the one hand, ASD has been previously suggested to serve as a vulnerability marker for PTSD, whereas on the other hand PTSD may worsen certain ASD symptoms^26^. At the molecular level, both ASD and PTSD may share common underlying mechanisms, such as neuronal dysfunction, leading to certain cognitive and behavioral outcomes, including avoidance, anger and aggression^26^.

Beyond ASD, trauma may occur as a result of various life-threatening events such as combat, terrorist attacks, accidents, and natural disasters. Following the stressful event, most survivors of trauma naturally resume their normal lives. However, some trauma victims suffer from long-lasting stress reactions that may worsen over time, and could develop into PTSD^27^. The symptoms of PTSD often include nightmares, insomnia and emotional numbness, and can significantly disrupt daily life. PTSD is associated with physical and psychological symptoms such as depression, substance abuse, and impairments of cognitive abilities^27^. Individuals suffering from PTSD often have social difficulties that can lead to family problems and occupational instability^28^. Therefore, PTSD as a consequence of frightening war experience or terror is a societal unmet need and is of high prevalence.

The behavioral manifestation of acute and chronic changes induced by stress in brain structure and function is thought to be represented by PTSD symptoms^27^. In this respect, two critical neurochemical systems in the stress response include cortisol and norepinephrine, serving as part of the feedback loop, which is important for the appropriate function of the hypothalamic-pituitary-adrenal (HPA) axis^27^. Importantly, a large body of evidence suggests that the HPA axis is tightly regulated by VIP and PACAP^29^.

Here, we extended our previous in-depth characterization of the *Adnp*-haploinsufficient mouse model, as a potential predictor of effects in humans subjected to stressful conditions. Specifically, we sought to determine the effect of the *Adnp* genotype on behavioral consequences following stress/trauma. Furthermore, given the known role of the ADNP-regulator PACAP in stress conditions^15,30,31^, its efficacy as a potential therapy was investigated, when applied as a preventative measure. PACAP administration also presents an ease-of-use advantage, as it can be provided as an intranasal brain-available native peptide^32^. Results showed impairments in stress-challenged *Adnp*^+/−^ mice (compared with non-challenged control animals, and stress-challenged *Adnp*^+/+^ mice) in the novel object recognition, social recognition and social memory tests, significantly improving by PACAP pre-treatment. Odor discrimination test revealed that the affected olfaction in the traumatized (challenged) mice was partially restored by PACAP. The assessment of anxiety-related behavior in the open field and elevated plus maze (EPM) showed that challenged mice exhibited altered behavior, normalized by PACAP. Splenic *Adnp* gene expression was found to be regulated by PACAP, with significant sex differences and a regulation dependent on *Adnp* gene dosage. Furthermore, splenic *Adnp* expression and plasma cortisol levels were positively correlated with cognition and anxiety-related behavior in mice. Complementing these findings and further extending to humans, lymphocytic *Adnp* gene expression was found to positively correlate with stress and salivary cortisol levels in subjects exposed to experimental stressful conditions, thus providing an additional translational dimension to our study.

Taken together, our results suggest a key role for ADNP in the stress response. This finding can be explained by the substantial correlation between *ADNP* levels and stress, as observed in the current study. Individuals expressing low *ADNP* transcript levels may exhibit a worse response to stressful/traumatic events, which can be significantly ameliorated by PACAP treatment, thus proving ADNP’s role in this context.

## Materials and methods

### Experimental Design

Animal group sizes were determined in a pilot study, and animals were randomly allocated into experimental groups before the experiment. The designation ‘N.Ch.’ in the relevant graphs represents non-challenged groups, whereas the designation ‘+Ch.’ represents stress-challenged groups. Blinded experienced researchers performed independently the different methodologies described in the manuscript, and repeated these successfully, thus substantiating the results. Technical replicates were used for gene expression analysis, whereas biological replicates were used for all the *in vivo* procedures described in the manuscript, including the correlations performed in animals and humans. Outlier values were determined and excluded by Grubbs’ test. The exact experimental group allocations are detailed in the Supplemental Materials and methods (Table S1) and are included for each figure panel presenting multiple samples.

### Animals

The *Adnp*^+/−^ mice, on a mixed C57BL and 129/Sv background, were previously described^3,4,21^. For continuous breeding, an ICR outbred mouse line was used^21,22^. Animals were housed in a 12-h light/12-h dark cycle animal facility, with free access to rodent chow and water. Genotyping was performed by Transnetyx (Memphis, TN, USA). For all experimental procedures, 9–12-month-old mice were treated twice daily with PACAP for one month. Then, a stress challenge was applied by 48 h of solitude in clean cage with overall dimensions of 32 × 18 × 12 cm (L × W × H) under constant bright illumination (~500 lux).

### Peptide synthesis and PACAP treatment

The PACAP38 peptide containing 38 residues HSDGIFTDSYSRYRKQMAVKKYLAAVLGKR-YKQRVKNK (Modifications: Lys-38 = C-terminal amide) was customarily synthesized by Hay Laboratories, Israel. For further detailed description of the peptide synthesis and treatment, please see supplemental materials and methods.

### Behavioral measurements and gene expression analysis in the *Adnp*^+/−^ mice

For detailed description of the social approach task, odor discrimination, object recognition test, open field, elevated plus maze, as well as gene expression analysis, please see supplemental materials and methods.

### Plasma Cortisol preparation

9–12-month-old mouse plasma was prepared from whole blood samples, centrifuged in Eppendorf Centrifuge 5417R at 6,000 RPM, 4 °C for 6 min. Then, plasma samples were further analyzed for cortisol using the IMMULITE^®^ 2000 Immunoassay System according to manufacturer’s protocol (IMMULITE^®^ 2000 Cortisol, Cat. No. L2KCO2/L2KCO6).

### Statistical Analysis

Results are presented as means ± standard error of the mean (SEM). Data were checked for normal distribution by normality test. For two different categorical independent variables, two-way analysis of variance (ANOVA) or two-way repeated measures ANOVA followed by Tukey post hoc or Bonferroni’s means separation methods were performed. Unpaired student’s *t*-test or Mann–Whitney U test analyses were performed when needed. *P* values smaller than 0.05 were considered significant. All tests were two-tailed. For *in vivo* procedures and gene expression analysis, outlier values were excluded using the Graphpad outlier calculator (https://graphpad.com/quickcalcs/Grubbs1.cfm). All statistical analyses were conducted using either SigmaPlot software version 11 Inc. for Windows (Chicago, IL, USA), or GraphPad Prism versions 5 & 6 Inc. for Windows (La Jolla, CA, USA).

### Study approval

All procedures involving animals were conducted under the supervision and approval of the Animal Care and Ethics Committee of Tel Aviv University and the Israeli Ministry of Health (M-15-059). All procedures involving human subjects were approved by the Tel Aviv Sourasky Medical Center (TASMC) Ethical Committee, and all participants provided written informed consent, approved by the ethical committee and conformed to the Code of Ethics of the World Medical Association (Helsinki Declaration).

## Results

### Stress worsens the social traits of *Adnp*^+/−^ mice, whereas *Adnp*^+/+^ mice are resistant, and treatment with PACAP normalizes the deficient mice

**Fig. 1:**
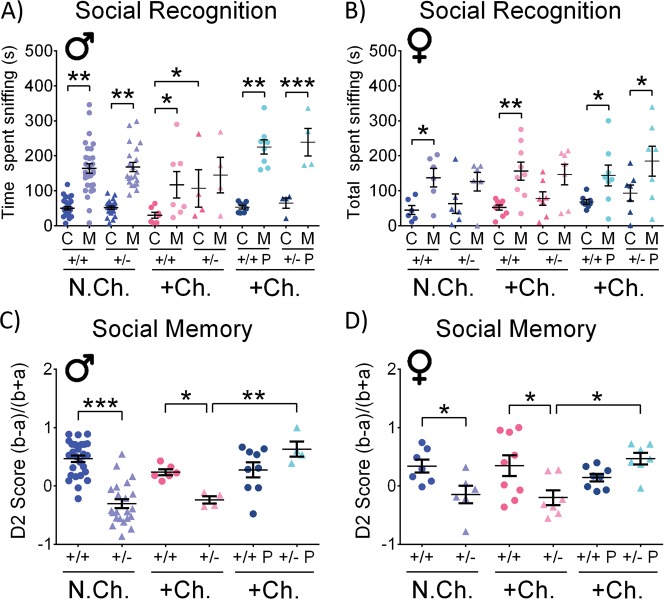
Stress-challenge worsens the social interaction abilities of *Adnp*^+/−^ mice, without affecting *Adnp*^+/+^ controls: PACAP treatment improves. Two-way ANOVA or two-way ANOVA repeated measures with Tukey post hoc test were performed (males (M): N.Ch. *Adnp*^+/+^ *N* = 28–29; N.Ch. *Adnp*^+/−^ *N* = 21–22; +Ch. *Adnp*^+/+^ *N* = 6–7; +Ch. *Adnp*^+/−^ *N* = 4; +Ch. *Adnp*^+/+^ PACAP *N* = 8–9; +Ch. *Adnp*^+/−^ PACAP *N* = 4; females (F): N.Ch. *Adnp*^+/+^ *N* = 6–7; N.Ch. *Adnp*^+/−^ *N* = 6; +Ch. *Adnp*^+/+^ *N* = 9; +Ch. *Adnp*^+/−^ *N* = 7; +Ch. *Adnp*^+/+^ PACAP *N* = 8; +Ch. *Adnp*^+/−^ PACAP *N* = 7). For social recognition, significant differences between sniffing time of the cup (C) and mouse (M) are described. **a** Stressed-challenged males: main effects for group (F(4,20) = 3.464, *p* = 0.026) and sniffed item (F(1,20) = 20.076, *p* < 0.001) were found, with differences in *Adnp*^+/+^ (*p < 0.05), and PACAP-treated *Adnp*^+/+^ (***p < 0.001), and *Adnp*^+/−^ mice (**p < 0.01). Vehicle-treated males: main effect for sniffed item was found (F(1,16) = 7.505, *p* = 0.015), with differences in non-challenged *Adnp*^+/+^ (**p < 0.01) and challenged *Adnp*^+/+^ mice (*p < 0.05). For empty cup sniffing times, main genotype effect was found (F(1,19) = 4.706, *p* = 0.043), with significant difference between challenged *Adnp*^+/+^ and *Adnp*^+/−^ mice (*p < 0.05). **b** Stressed-challenged females: main effect for sniffed item was found (F(1,27) = 21.931, *p* < 0.001), with differences in *Adnp*^+/+^ (**p < 0.01), and PACAP-treated *Adnp*^+/+^ (*p < 0.05), and *Adnp*^+/−^ mice (*p < 0.05). Vehicle-treated females: main effect for sniffed item was found (F(1,24) = 22.325, *p* < 0.001), with differences in non-challenged and challenged *Adnp*^+/+^ mice (*p < 0.05). **c** Stressed-challenged males: main treatment (F(1,19) = 13.257, *p* = 0.002) and interaction (F(1,19) = 11.072, *p* = 0.004) effects were found, with differences between challenged PACAP- and vehicle-treated *Adnp*^+/−^ mice (***p < 0.001), and *Adnp*^+/+^ vs. *Adnp*^+/−^ mice (*p < 0.05). Vehicle-treated males: main genotype effect (F(1,56) = 33.535, *p* < 0.001) was found, with differences between non-challenged *Adnp*^+/+^ and *Adnp*^+/−^ mice (***p < 0.001), and challenged *Adnp*^+/+^ vs. *Adnp*^+/−^ mice (*p < 0.05). **d** Stressed-challenged females: main interaction effect was found (F(1,27) = 7.507, *p* = 0.011), with differences between challenged PACAP- and vehicle-treated *Adnp*^+/−^ mice (*p < 0.05), and challenged *Adnp*^+/+^ vs. *Adnp*^+/−^ mice (*p < 0.05). Vehicle-treated females: main genotype effect was found (F(1,25) = 11.477, *p* = 0.002), with differences between non-challenged *Adnp*^+/+^ and *Adnp*^+/−^ mice (*p < 0.05), and challenged *Adnp*^+/+^ vs. *Adnp*^+/−^ mice (*p < 0.05)

Nine- to twelve-month-old mice were subjected to a twice daily PACAP treatment for one month, followed by 48 h of solitude in clean cage under constant bright illumination (stressful challenging conditions). In the social recognition test, the behavioral phenotype of stress-challenged male *Adnp*^+/−^ mice was exacerbated, compared with their control group, as the challenged animals did not have any significant preference to either cup or mouse, contrasting the non-challenged mice and the challenged *Adnp*^+/+^ mice preference of mice over objects (Fig. 1a). Thus, a significant increase in cup sniffing time was observed in the challenged *Adnp*^+/−^ group, in comparison to challenged *Adnp*^+/+^ mice (*p < 0.05). Treatment with PACAP normalized the stress-challenged *Adnp*^+/−^ male mice (Fig. 1a). In females, as previously observed, non-challenged *Adnp*^+/−^ mice showed no significant preference for mice over objects^7^, and here, this behavior was not altered with stress. However, similar to males (Fig. 1a), PACAP treatment resulted in stressed *Adnp*^+/−^ female mice preferring mice over objects, (Fig. 1b). The social memory test results depicted in Fig. 1c, d showed a substantial effect of the *Adnp* genotype in both sexes, with the *Adnp*-haploinsufficient mice displaying reduced interest toward an unfamiliar mouse over a familiar mouse, compared with *Adnp*-intact mice (***p < 0.001 in males, *p < 0.05 in females). These results also correlate previous findings from our laboratory^7,21,22^. The stress challenge did not affect social memory and the difference between stress-challenged *Adnp*^+/−^ mice, exhibiting a significantly reduced interest in the non-familiar mouse, compared with challenged *Adnp*^+/+^ mice was preserved (Fig. 1c, d, *p < 0.05). PACAP treatment had a dramatic positive effect on the stress-challenged *Adnp*^*+/−*^ mice, with significant improvement in the social memory to the control levels (Fig. 1c, d, **p < 0.01 in males, *p < 0.05 in females).

As social familiarity develops mainly through the mechanism of olfactory cues^33^, the effects of stress and PACAP on odor discrimination were investigated. Stress-challenge led to odor discrimination abolishment in *Adnp*^+/+^ and *Adnp*^+/−^ mice (almond discrimination in males, vanilla discrimination in females), whereas pre-treatment with PACAP protected odor discrimination (Fig. S1A–D).

### Stress challenge impairs the cognitive state of both male and female *Adnp*^+/−^ mice, compared with *Adnp*^+/+^ counterparts: PACAP pre-treatment protects

**Fig. 2:**
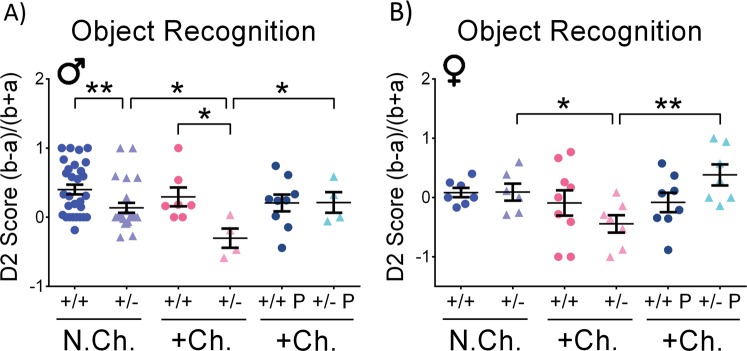
*Adnp*^+/+^ mice display intact cognitive phenotype under stress-challenge, as opposed to *Adnp*^+/−^ mice: PACAP treatment ameliorates. Two-way ANOVA with Tukey post hoc test was performed (males (M): N.Ch. *Adnp*^+/+^ *N* = 29; N.Ch. *Adnp*^+/−^ *N* = 24; +Ch. *Adnp*^+/+^ *N* = 7; +Ch. *Adnp*^+/−^ *N* = 4; +Ch. *Adnp*^+/+^ PACAP *N* = 9; +Ch. *Adnp*^+/−^ PACAP *N* = 4; females (F): N.Ch. *Adnp*^+/+^ *N* = 7; N.Ch. *Adnp*^+/−^ *N* = 6; +Ch. *Adnp*^+/+^ *N* = 9; +Ch. *Adnp*^+/−^ *N* = 7; +Ch. *Adnp*^+/+^ PACAP *N* = 8; +Ch. *Adnp*^+/−^ PACAP *N* = 7). **a** In stress-challenged male mice, main genotype (F(1,20) = 4.444, *p* = 0.048) and interaction (F(1,20) = 4.628, *p* = 0.044) effects were found, with significant differences between PACAP- and vehicle-treated *Adnp*^+/−^ mice (*p < 0.05), and *Adnp*^+/+^ vs. *Adnp*^+/−^ mice (**p < 0.01). In vehicle-treated male mice, main genotype (F(1,60) = 12.085, *p* < 0.001) and challenge (F(1,60) = 4.873, *p* = 0.031) effects were found, with significant differences between non-challenged *Adnp*^+/+^ and *Adnp*^+/−^ mice (*p < 0.05), and challenged vs. non-challenged *Adnp*^+/−^ mice (*p < 0.05). **b** In stress-challenged female mice, main treatment effect was found (F(1,27) = 4.392, *p* = 0.046), with a significant difference between PACAP- and vehicle-treated *Adnp*^+/−^ mice (**p < 0.01). In vehicle-treated female mice, main challenge effect was found (F(1,25) = 4.419, *p* = 0.046), with a significant difference between stress-challenged and non-stressed *Adnp*^+/−^ mice (*p < 0.05)

The object recognition procedure takes advantage of an animal’s tendency to approach and explore novelty, differentiating familiar from novel objects. Mouse performance in the object recognition memory task is presented in Fig. 2a, b. The fraction of time spent with the novel object in relation to the total time spent sniffing both novel and familiar objects (D2) was analyzed. There were no significant differences in the short retention choice phase (3 h after habituation, data not shown). However, in the long retention choice phase (24 h after habituation), a significant impact of the *Adnp* genotype was observed in non-challenged males, with *Adnp*^+/−^ mice displaying reduced D2 score, compared with *Adnp*^+/+^ mice (**p < 0.01), as previously described^21^. Interestingly, stress-challenge exacerbated the behavioral phenotype of *Adnp*^+/−^ mice in both sexes, whereas *Adnp*^+/+^ mice showed intact behavior (*p < 0.05). Importantly, for both males and females, treatment with PACAP significantly protected (normalized) long-term memory in stress-challenged *Adnp*^+/−^ mice (*p < 0.05 for males, **p < 0.01 for females).

### Splenic *Adnp* gene expression is differentially regulated by PACAP in a sex- and genotype-dependent manner and positively correlates with cognition in non-challenged males

In humans, lymphocytic/systemic ADNP expression was previously found to correlate with inflammation^34^, mental disease state^35^, as well as intelligence^36^. In the current study, to provide a more substantial link between potential peripheral markers at the molecular level and the *in vivo* behavioral outcomes, we further looked into possible correlations between mouse splenic *Adnp* expression and cognitive scores.

**Fig. 3:**
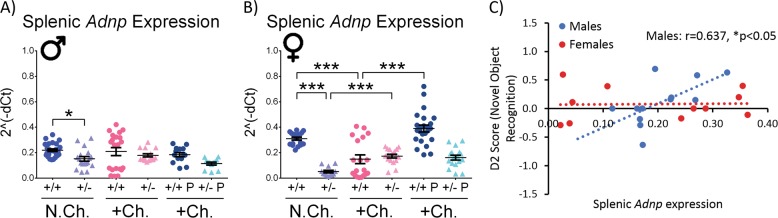
Splenic *Adnp* gene expression is regulated in a sex-dependent manner, as well as by PACAP treatment in females, with cognition positively correlating splenic *Adnp* expression. Two-way ANOVA with Tukey post hoc test was performed (males (M): N.Ch. *Adnp*^+/+^ *N* = 12–26; N.Ch. *Adnp*^+/−^ *N* = 12–18; +Ch. *Adnp*^+/+^ *N* = 21; +Ch. *Adnp*^+/−^ *N* = 15; +Ch. *Adnp*^+/+^ PACAP *N* = 14; +Ch. *Adnp*^+/−^ PACAP *N* = 12; females (F): N.Ch. *Adnp*^+/+^ *N* = 18; N.Ch. *Adnp*^+/−^ *N* = 12; +Ch. *Adnp*^+/+^ *N* = 19; +Ch. *Adnp*^+/−^ *N* = 17; +Ch. *Adnp*^+/+^ PACAP *N* = 24; +Ch. *Adnp*^+/−^ PACAP *N* = 17). For gene expression analysis, results are presented as 2^−ΔCT^, normalized to *Hprt*. For correlation testing, if both plotted data sets were normally distributed, a Pearson’s correlation analysis was performed. If at least one of the data sets was not normally distributed, a Spearman’s correlation was performed. **a** Splenic *Adnp* expression in vehicle-treated males, significant main genotype effect (F(1,76) = 5.664, *p* = 0.020) was found, with significant differences between non-challenged *Adnp*^+/+^ and *Adnp*^+/−^ mice (*p < 0.05). **b** Splenic *Adnp* expression in stressed-challenged females: main genotype (F(1,73) = 15.336, *p* < 0.001), treatment (F(1,73) = 19.029, *p* < 0.001), and interaction (F(1,73) = 23.179, *p* < 0.001) effects were found, with significant differences between challenged *Adnp*^+/+^ and *Adnp*^+/−^ mice (***p < 0.001), as well as challenged PACAP- vs. vehicle-treated *Adnp*^+/+^ mice (***p < 0.001). In vehicle-treated females, significant main genotype (F(1,62) = 26.464, *p* < 0.001) and interaction (F(1,62) = 38.150, *p* < 0.001) effects was found, with significant differences between non-challenged *Adnp*^+/+^ and *Adnp*^+/−^ mice (***p < 0.001), challenged vs. non-challenged *Adnp*^+/−^ mice (***p < 0.001), and challenged vs. non-challenged *Adnp*^+/+^ mice (***p < 0.001). **c** In non-challenged males (*N* = 12), splenic *Adnp* gene expression was positively correlated with the D2 score in the novel object recognition test (Pearson’s correlation, *r* = 0.637, *p < 0.05)

Firstly, in order to characterize ADNP as a potential peripheral biomarker for the behavioral outcome of stressful situations, the expression level of splenic *Adnp* was measured in all the experimental groups in our study, using qRT-PCR (Fig. 3a, b). The measurements assessed genotype, sex, treatment and stress effects. *Hprt* was used as a validated reference transcript^7^. Results supported the *Adnp*^+/−^ mouse model with decreased *Adnp* expression in the non-challenged *Adnp*^+/−^ groups, compared with *Adnp*^+/+^ groups in both sexes (*p < 0.05 in males, ***p < 0.001 in females). Stress-challenge had a genotype- and sex-dependent effect, with *Adnp* expression decreasing in *Adnp*^+/+^ females, while increasing in *Adnp*^+/−^ female mice (***p < 0.001). When treating with PACAP, *Adnp* expression was increased only in the stress-challenged *Adnp*^+/+^ females (***p < 0.001), indicating a sex-, stress-, gene dosage-dependent regulation and pleiotropic effects for PACAP.

Correlating *Adnp* expression and cognition, a significant positive correlation was found in non-challenged males between splenic *Adnp* expression and the D2 score of the novel object recognition test, measuring cognition (Fig. 3c, *p < 0.05).

To further understand the relations between ADNP and the PACAP regulatory system expression (including the PACAP binding PAC1, VPAC1, and VPAC2 receptors), we examined a previous complete RNA-seq analysis performed in the *Adnp*^+/−^ mouse model, using submitted data to GEO (accession number GSE72664)^22^. For *Adcyap1* gene encoding PACAP in 1-month-old male mice, expression was decreased in *Adnp*^+/−^ compared with *Adnp*^+/+^ mice (*p < 0.05, Fig. S2A). In *Adnp*^+/+^ male mice, *Adcyap1* gene expression was decreased in 5-month- compared with 1-month-old mice (**p < 0.01, Fig. S2A). No differences were detected in female mice (Fig. S2B). For the *Adcyap1r1* gene encoding PACAP’s specific receptor PAC1 in males, expression was decreased in 5-month-old *Adnp*^+/+^ (***p < 0.001, Fig. S2C) and *Adnp*^+/−^ mice (**p < 0.01, Fig. S2C), compared with 1-month-old mice of both genotypes. Thus, in males, PACAP and the PAC1 receptor expression are decreased with *Adnp* deficiency and with maturation/aging (Fig. S2).

In female mice, *Adcyap1r1* gene expression was decreased in 5-month-old *Adnp*^+/−^ mice, compared with *Adnp*^+/+^ mice (**p < 0.01, Fig. S2D), coupled with an age-dependent decrease for *Adnp*^+/−^ mice (***p < 0.001, Fig. S2D).

When looking at the genes encoding VIP receptors, VPAC1 and VPAC2 (*Vipr1* and *Vipr2*), significant changes were observed in female mice and no differences were found in male mice (Fig. S3A–D). Specifically, *Vipr1* gene expression was decreased in 1-month-old *Adnp*^+/−^ mice, compared with *Adnp*^+/+^ mice (*p < 0.05, Fig. S3B). *Vipr2* expression was increased in 1-month-old *Adnp*^+/−^ mice and decreased in 5-month-old *Adnp*^+/−^, when compared with *Adnp*^+/+^ mice of the same age (***p < 0.001, Fig. S3D). *Vipr2* gene expression was also increased in 5-month- compared with 1-month-old *Adnp*^+/+^ mice, whereas in *Adnp*^+/−^ mice it was decreased with age (***p < 0.001, Fig. S3D).

Thus, there seems to be an intricate closed loop regulation of ADNP and the PACAP system with sex and age dependency, and with significant changes of PAC1 expression in males and VPAC2 expression in females.

### Stress-challenged female *Adnp*^+/−^ mice exhibit altered anxiety-related behavior, normalized by PACAP treatment and positively correlating plasma cortisol levels

**Fig. 4:**
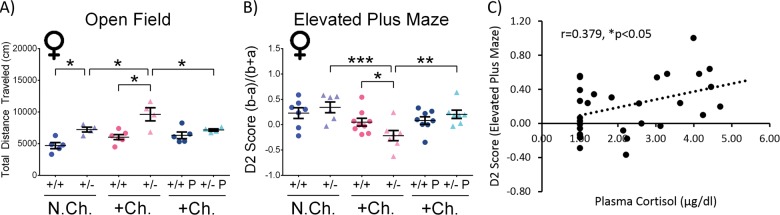
Stress-challenged female *Adnp*^+/−^ mice exhibit altered behavior in the open field and elevated plus maze, with PACAP ameliorating and anxiety-related behavior correlating plasma cortisol levels. Two-way ANOVA with Tukey post hoc test was performed (females (M): N.Ch. *Adnp*^+/+^ *N* = 5–7; N.Ch. *Adnp*^+/−^ *N* = 4–6; +Ch. *Adnp*^+/+^ *N* = 6–9; +Ch. *Adnp*^+/−^ *N* = 4–7; +Ch. *Adnp*^+/+^ PACAP *N* = 5–8; +Ch. *Adnp*^+/−^ PACAP *N* = 4–7). **a** *Adnp*^+/−^ female male mice exhibit markedly increased total distance traveled in the open field apparatus, further increased by stress-challenge and reduced by PACAP treatment. In stress-challenged females, main effects for genotype (F(1,15) = 14.358, *p* = 0.002) and interaction (F(1,15) = 5.541, *p* = 0.033) were found, with significant differences between PACAP- and vehicle-treated *Adnp*^+/−^ mice (*p < 0.05), and *Adnp*^+/+^ vs. *Adnp*^+/−^ mice (***p < 0.001). In vehicle-treated females, significant main genotype (F(1,15) = 26.946, *p* < 0.001) and treatment (F(1,15) = 10.137, *p* = 0.006) effects were found, with significant differences between non-challenged *Adnp*^+/+^ and *Adnp*^+/−^ mice (*p < 0.05), challenged vs. non-challenged *Adnp*^+/−^ mice (*p < 0.05), and challenged *Adnp*^+/+^ vs. *Adnp*^+/−^ mice (***p < 0.001). **b** In female mice, stress-challenged *Adnp*^+/−^ mice spent substantially more time in the open arms compared with non-challenged *Adnp*^+/−^ mice, challenged *Adnp*^+/+^ mice, and challenged PACAP-treated *Adnp*^+/−^ mice. In stress-challenged females, main effects for treatment (F(1,27) = 7.823, *p* = 0.009) and interaction (F(1,27) = 5.779, *p* = 0.023) were found, with a significant difference between PACAP- and vehicle-treated *Adnp*^+/−^ mice (**p < 0.01), as well as *Adnp*^+/+^ vs. *Adnp*^+/−^ mice (*p < 0.05). In vehicle-treated females, a main effect for stress-challenge was found (F(1,25) = 15.297, *p* < 0.001), with a significant difference between non-challenged and challenged *Adnp*^+/−^ mice (***p < 0.001), as well as challenged *Adnp*^+/+^ vs. *Adnp*^+/−^ mice (*p < 0.05). **c** For all experimental groups in the study (total *N* = 30), plasma cortisol levels were positively correlated with the D2 score in the elevated plus maze test (Spearman’s correlation, *r* = 0.379, *p < 0.05)

To further characterize the stress response in the treated mice, anxiety-related behavior was evaluated in the open field and the EPM behavioral tests. In the open field (Fig. 4a), the total distance traveled/locomotor activity was measured in all the experimental groups. This parameter has been previously found to be relevant to positive symptoms of schizophrenia, and general psychotic behavior^37^. In the current study, non-challenged *Adnp*^+/−^ female mice covered significantly longer distance, compared with *Adnp*^+/+^ female mice (*p < 0.05). Stress-challenge exacerbated this behavior, causing a further significant increase in the total distance traveled by the *Adnp*^+/−^ females, in comparison with their non-challenged counterparts, and stress-challenged *Adnp*^+/+^ mice (*p < 0.05). PACAP treatment in stress-challenged *Adnp*^+/−^ mice significantly decreased the total distance traveled (*p < 0.05).

An additional evaluation of anxiety-related behavior was performed by the EPM test (Fig. 4b). In females, stress-challenged *Adnp*^+/−^ mice spent significantly more time in the open arms, compared with non-challenged *Adnp*^+/−^ (***p < 0.001), and challenged *Adnp*^+/+^ mice (*p < 0.05), indicative of a possible altered anxiety-related/increased risky behavior. PACAP treatment normalized this phenotype (**p < 0.01). As opposed to females, males did not exhibit any significant changes in association with the *Adnp* genotype or with PACAP treatment in neither the open field, nor the EPM behavioral tests (Fig. S4).

It should be noted that cortisol is closely linked with stress, acting as a key player in the body’s stress response and often measured as an indicator of stress^38^. Here, when examining animals in all the experimental groups, a significant positive correlation was observed between plasma cortisol levels and the D2 score in the EPM test, measuring anxiety-related behavior (Fig. 4c, *p < 0.05).

### Human *ADNP* expression positively correlates with stress and plasma cortisol levels, thus serving as a potential marker of stress response

**Fig. 5:**
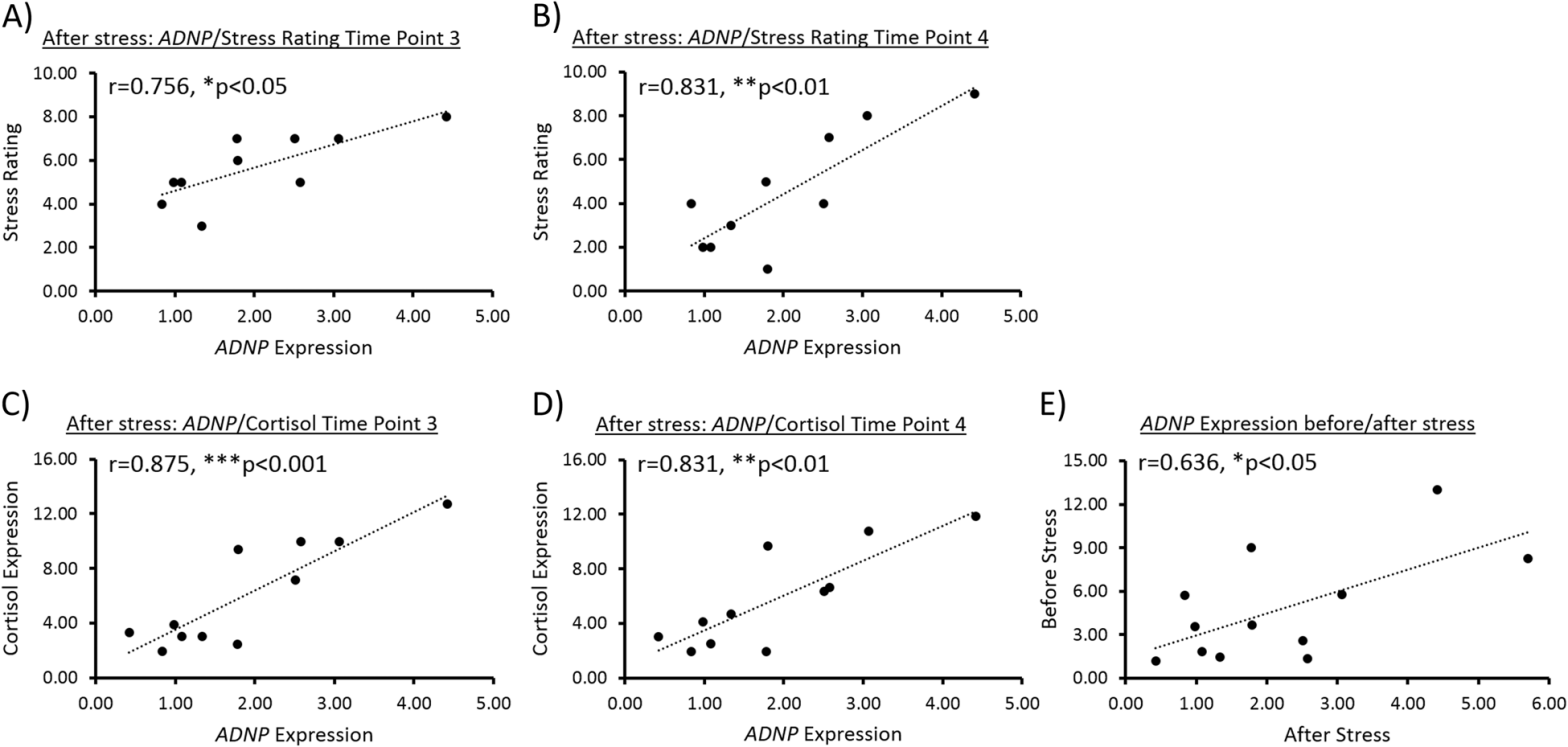
Human *ADNP* expression is positively correlated with stress and salivary cortisol levels. In a human cohort (*N* = 10–11 subjects), data sets of either stress rating (on a 9-point Likert scale) or salivary cortisol levels were plotted against lymphocytic ADNP gene expression level. In case both data sets were normally distributed, data were checked for Pearson’s correlation. If at least one of the data sets was not normally distributed, data were checked for Spearman’s correlation. **a** Lymphocytic *ADNP* gene expression was positively correlated with the stress rating, right after the end of the stress task (Pearson’s correlation, *r* = 0.757, *p < 0.05). **b** Lymphocytic *ADNP* gene expression was positively correlated with the stress rating, 20 min after the end of the stress task (Pearson’s correlation, *r* = 0.832, *p < 0.01). **c** Lymphocytic *ADNP* gene expression was positively correlated with salivary cortisol levels, right after the end of the stress task (Spearman’s correlation, *r* = 0.785, **p < 0.01). **d** Lymphocytic *ADNP* gene expression was positively correlated with salivary cortisol levels, 20 min after the end of the stress task (Pearson’s correlation, *r* = 0.832, **p < 0.01). **e** Lymphocytic *ADNP* gene expression level before the stress task was positively correlated with its level after the stress task (Pearson’s correlation, *r* = 0.636, **p < 0.05)

Given our results in animals, and in order to deliver further evidence to the role of ADNP in the stress response, we aimed at providing an additional translational aspect to our study, by looking at a human cohort. For this purpose, we examined behavioral and physiological data, as well as lymphocytic RNA samples, previously collected, described and extracted from healthy male IDF soldiers, before and after being subjected to experimental stressful conditions^38^. Strikingly, significant positive correlations were found between *ADNP* expression and stress rating (on a 9-point Likert scale) at two time points after exposure to stress (Fig. 5a, b, *p < 0.05 right after the end of the stress task - time point 3, **p < 0.01 20 min after the end of the stress task - time point 4). Additional positive correlations were found between *ADNP* expression and salivary cortisol levels at the same time points described above (Fig. 5c, d). No significant correlations were observed between *ADNP* and either stress rating or salivary cortisol levels before inducing the stressful conditions (Fig. S5A–D). Furthermore, stress recovery/sustainment values were calculated to evaluate traces of stress observed in the reports of participants in the human cohort, as previously described^38^. Changes in the ratings were calculated 20-min after stress induction ended, as compared with the ratings obtained immediately after the stress task. A decrease in the reported post-stress experience indicated more effective emotion regulation processing. Therefore, a recovery of subjective stress was regarded as a reduction in stress ratings, whereas a sustainment of subjective stress was regarded as a lack of reduction (Δ > = 0)^38^. Although insignificant, lymphocytic *ADNP* gene expression presented a trend of positive correlation with recovery/sustainment values, indicating a possible marker of stress response in stress-sustained subjects (Fig. S5E). Interestingly, *ADNP* expression levels before stress introduction were positively correlated with those after exposure to stress (*p < 0.05), thus implying that the higher the *ADNP* levels were before stress, the higher these remained after being exposed to it (Fig. 5e).

## Discussion

In the current study, the aspects of anxiety levels, as well as cognition and social activity were examined *in vivo* in the unique mouse model of *Adnp* haploinsufficiency, compared with normal mice exposed to stressful conditions of 48 h of constant bright illumination and solitude. Our results indicate that *Adnp* deficiency in mice renders the animals more susceptible to stress, and that this condition may be ameliorated by exogenous PACAP administration. In this regard, higher expression levels of the ADNP-regulator, PACAP, and its specific PAC1 receptor have been previously associated with stress response^39^. This was found in several stress- and anxiety-related brain regions, including the hypothalamus, hippocampus, amygdala, and the bed nucleus of the stria terminalis (BNST)^40–42^. Interestingly, ADNP was also found to be expressed in these regions^4,21,43^. When looking at *in vivo* behavioral outcomes, an anxiety-like behavior was enhanced with PACAP infusion into the BNST, producing a long-lasting anxiogenic behavioral response^39^. Importantly, the PAC1 receptor was previously shown to co-localize with ADNP in mouse brains, and since ADNP is regulated by PACAP, this may further suggest and strengthen the idea of ADNP being involved in the stress response as well^44^.

Furthermore, PACAP was previously found to progressively decline throughout life offering less protective effects, whereas the overexpression of PACAP reversed this process, producing a young phenotype^45,46^. Age-related PACAP decline leads to pathophysiological changes, including vulnerability to stressors, increased apoptosis, enhanced sensitivity to oxidative stress, pro-inflammatory environment and degeneration with amyloid deposits^47^, also described in the *Adnp*-deficient mice, presenting increased age-dependent neurodegeneration and tauopathy^4,7^. It should be noted that similarly to the *Adnp*-haploinsufficient mouse model, PACAP-deficient mice were found to have slower weight gain during the first weeks of development, coupled with general slower neurobehavioral development^7,48^. Interestingly, PACAP was also found to affect dentition in old and young mice^49,50^, and ADNP expression was associated with tooth eruption in mice and children^51^. These findings provide additional evidence to the existing important link between ADNP and PACAP^7,48^. Finally, at the mechanistic level, both ADNP and PACAP are linked to cytoskeletal health, providing neurotrophic and neuroprotective effects^7,52^.

Sexual dichotomy was observed in the social recognition test, where the stress affected only the *Adnp*^+/−^ male mice (showing a similar interest in objects and mice that implies of an “autistic behavior”) and not the female mice, as the genotype effect was observed in females not exposed to stress as well. In contrast, novel object recognition was impaired in non-stressed *Adnp*^+/−^ males, and not in the counterpart female cohort, with both sexes significantly affected by stress and ameliorated by PACAP treatment. Social memory deficits as a consequence of the *Adnp*^+/−^ genotype were not significantly altered by stress, and were ameliorated by PACAP treatment. In the odor discrimination test, stress-challenge affected olfaction in mice of both genotypes and sexes, whereas treatment with PACAP restored it. These results suggest a significant impact of the *Adnp* genotype on cognitive/social traits when exposed to stressful conditions, in a sex-dependent manner, and PACAP as potential preventative measure/therapeutics.

As indicated above, in the novel object recognition, non-challenged male *Adnp*^+/+^ mice preferred novel objects, compared with *Adnp*^+/−^ mice, exhibiting indifference or familiarity preference. In females, no significant differences were observed between non-challenged *Adnp*^+/+^ and *Adnp*^+/−^ mice. In the current study, the tested mice were 9–12 months of age. This result in 12-month-old females correlates previous findings in 7-month-old female mice^21^, but contradicts data indicating object memory deficits in young 3-month-old female *Adnp*^+/−^ mice^7^. These different outcomes may be related to an age effect, indicating a possible age-related deterioration of performance in female *Adnp*^+/+^ mice in the object recognition test. Since PACAP regulates ADNP, its decline with age may also be linked^47^. When looking at sex differences in 7-month-old mice, we (IG) previously showed a 2-fold decreased *Adnp* expression in the female *Adnp*^+/+^ hippocampus, compared to males, with no significant sex differences in *Adnp*^+/−^ mice^21^. In human postmortem hippocampi, similar sex differences were found (25% less *ADNP* in females vs. males^21^). These findings are coupled to estrus cycle regulation of *Adnp* in the mouse hypothalamus^43^. Following stress-challenge, *Adnp*^+/−^ mice exhibited significant impairments, compared with either non-challenged *Adnp*^+/−^ counterparts or challenged *Adnp*^+/+^ control mice, and with PACAP treatment ameliorating these impairments. Interestingly, a significant correlation was also found between cognitive behavioral results and splenic *Adnp* gene expression. In non-challenged male mice, the D2 score in the novel object recognition test was positively correlated with splenic *Adnp* expression. This result is supported by the previous finding of the blood-borne expression of ADNP correlating with premorbid intelligence, Alzheimer’s disease pathology and clinical stage^36^. Importantly, as an ADNP-regulator, PACAP serves as a potent α-secretase activator, previously found to slow down AD-like pathology in amyloid precursor protein-transgenic mice^53^.

Furthermore, *Adnp* gene expression in the spleen was found to be differentially regulated by PACAP in a sex- and genotype-dependent manner, suggesting a gene dosage-dependent regulation affected by stress-challenge. These results may also indicate ADNP as a potential peripheral biomarker of stress response. Thus, previous findings from our lab (IG) indicated various splenic genotype-specific gene expression changes, corrected by the ADNP-snippet, NAP, in *Adnp*-deficient mice, specifically *Adnp* transcript haploinsufficiency in the young, developing mouse^7^. However, the majority of splenic gene expression changes were found in 3-month-old “mature” mice, compared with 19- to 27-day-old “young” mice, suggesting an age-dependent regulation^7^, as in the case of age-related PACAP reduction^47^. In this respect, using RNA-seq data^22^, Fig. S2A–D showed an age-dependent reduction in PACAP expression in males, and in PAC1 gene expression in both sexes. This was coupled to a highly significant age- and genotype- (*Adnp*^+/+^ vs. *Adnp*^+/−^) related reduction in VPAC2 gene expression in female mice. Interestingly, duplications of the *Vipr2* gene were found to confer a significant risk for schizophrenia, thus suggestive of a differential emotional response in the *Adnp*^*+/−*^ female mice^54^.

Indeed, when looking into anxiety-related behavior, *Adnp*^+/−^ female mice exhibited a significantly increased total distance traveled, previously linked with psychotic behavior^37^, compared with *Adnp*^+/+^ mice. This behavior was further exacerbated with stress-challenge, and normalized by PACAP treatment. Similarly, in the EPM, stress-challenged *Adnp*^+/−^ female mice spent significantly more time in the open arms, thus exhibiting altered/risky behavior, compared with non-challenged *Adnp*^+/−^ and challenged *Adnp*^+/+^ mice. PACAP treatment normalized this behavior. The finding of stress-challenged *Adnp*^+/−^ mice spending more time in the open arms of the EPM apparatus may also be indicative of a reduced anxiety-like phenotype, previously found in PACAP- and PAC1 receptor-null mice, implying of PACAP’s role in stress mechanisms^39,55^. This is also consistent with the anxiogenic role of PACAP mentioned above^56–58^. Interestingly, previous findings obtained from a human cohort showed significant correlation between PTSD symptoms and the blood levels of the PACAP peptide containing 38 residues (PACAP38) specifically in females^14^. It should also be noted that females are known to be at a substantial higher risk for developing PTSD, compared with males^59,60^. This may be attributed to certain roles of sex hormones, mainly estrogen, in the disorder^61–63^. In addition, when looking at all the experimental groups in our study, the D2 score in the EPM test was positively correlated with plasma cortisol levels. Shown for the first time in the *Adnp*-haploinsufficient mouse model, this result supports the idea that higher cortisol levels allow the organism to cope better with stress, given the well-known role of cortisol as the “stress hormone”^27^, and that increased D2 score in the EPM indicates an increased stress level.

Lastly, our results were extended to humans, exhibiting striking positive correlations between peripheral lymphocytic *Adnp* gene expression and stress/salivary cortisol levels after exposure to stressful conditions. This finding complements a previous result showing significant positive correlation between stress rating and salivary cortisol levels in the same study^38^. Human peripheral blood mononuclear cells (PBMCs) express *ADNP*^34^, and circulating ADNP (human serum/plasma^36,64^) is correlated with increased cognitive function^36^. ADNP suppresses its own transcription^5,65^, and lymphocyte ADNP was found to be upregulated in Alzheimer’s disease and in schizophrenia patients in a sex-dependent manner^35,36^. ADNP contains cellular export and cellular uptake sequences^66^, and interacts in the cytoplasm with microtubule end binding (EB) proteins through its SxIP motif (found within its neuroprotective fragment NAPVSIPQ)^67^, and with MAP1-LC3 through several binding motifs^68^. The EB and LC3 interactions provide microtubule and autophagy protection and are increased in the presence of NAP^35^. Therefore, ADNP’s mechanism of action in lymphocytes probably involves an EB1-mediated microtubule pathway, previously found to induce autophagy and cytokine secretion in a LC3-dependent fashion^69^. Furthermore, other microtubule growing tip regulating proteins have been linked to the control of cytokine production/release in PBMCs^70^. In this respect, reduction in ADNP has been linked to increases in pro-inflammatory cytokines, with exogenous NAP suppressing the production of the pro-inflammatory cytokines TNF-α, IL-6, and IL-12 from murine macrophages and human PBMCs^34,71^. Importantly, PTSD has been associated with pro-inflammatory changes^72^, and PTSD symptoms were previously found to significantly correlate with PACAP38 blood levels in females^14^. The diagnosis of PTSD was also associated with the levels of PACAP38, with higher levels of it measured in the PTSD cohort^14^, correlating with our current study findings.

To summarize, in the present study, *Adnp*^+/−^ mice were affected more dramatically by stressful conditions, and responded more effectively to PACAP treatment than *Adnp*^+/+^ mice. In addition, significant sex differences were observed, with *Adnp*^+/−^ males more susceptible to stress in the object and social recognition tests, and the females more susceptible in the open field and EPM tests. These results suggest a significant impact of the *Adnp* genotype when exposed to stressful conditions, and PACAP as potential future preventative measure/therapeutics. Therefore, in connection with the correlation of blood ADNP levels with AD^36^, our results may suggest an additional correlation between ADNP levels and stressful conditions/PTSD. In such a case, low ADNP measures in the blood may serve as indication for a worse response to stressful events, which can be successfully ameliorated by PACAP pre-treatment. Altogether and for the first time to our knowledge, our results set ADNP as a potential marker of stress response, with low *ADNP* transcript expression levels indicating a worse response to stressful events, which can be successfully ameliorated by PACAP treatment. This could lay the foundations for ADNP as a possible biomarker, identifying people who are prone to suffer from post-traumatic stress or already suffering from its symptoms, as well as the intake of PACAP as a potential prevention measure. Future studies are planned to translate our promising results to humans.

## Supplementary information

Supplementary Materials
