# Supplementary material for: The autism-mutated ADNP plays a key role in stress response
Source: Transl Psychiatry. 2019 Sep 18;9:235. doi: 10.1038/s41398-019-0569-4 (PMC6751176; doi:10.1038/s41398-019-0569-4)
Supplement: Supplementary file 1 — Supplementary Materials [file 41398_2019_569_MOESM1_ESM.pdf]

Supplementary Materials:

The autism-mutated ADNP plays a key role in stress response

Shlomo Sragovich[1], Yarden Ziv[1], Sharon Vaisvaser[2], Noam Shomron[3], Talma Hendler[2,4] and Illana Gozes[1*]

[1]The Lily and Avraham Gildor Chair for the Investigation of Growth Factors; The Elton Laboratory for Neuroendocrinology; Department of Human Molecular Genetics and Biochemistry, Sackler Faculty of Medicine, Sagol School of Neuroscience and Adams Super Center for Brain Studies, Tel Aviv University, Tel Aviv 69978, Israel; [2]Functional Brain Center, Wohl Institute for Advanced Imaging, Sourasky Medical Center, Tel Aviv, Israel; [3]Sackler Faculty of Medicine, Tel Aviv University, Tel Aviv 69978, Israel; [4]School of Psychological Sciences, Sackler Faculty of Medicine, Sagol School of Neuroscience, Tel Aviv University, Tel Aviv 69978, Israel.

[*]**Corresponding author:**

Illana Gozes, Ph.D.; Professor of Clinical Biochemistry

The Lily and Avraham Gildor Chair for the Investigation of Growth Factors

Head, the Dr. Diana and Zelman Elton (Elbaum) Laboratory for Molecular Neuroendocrinology

Sackler Faculty of Medicine, Tel Aviv University

Tel Aviv 69978, Israel, Phone : 972-3-640-7240, Fax : 972-3-640-8541

E-mail : igozes@tauex.tau.ac.il

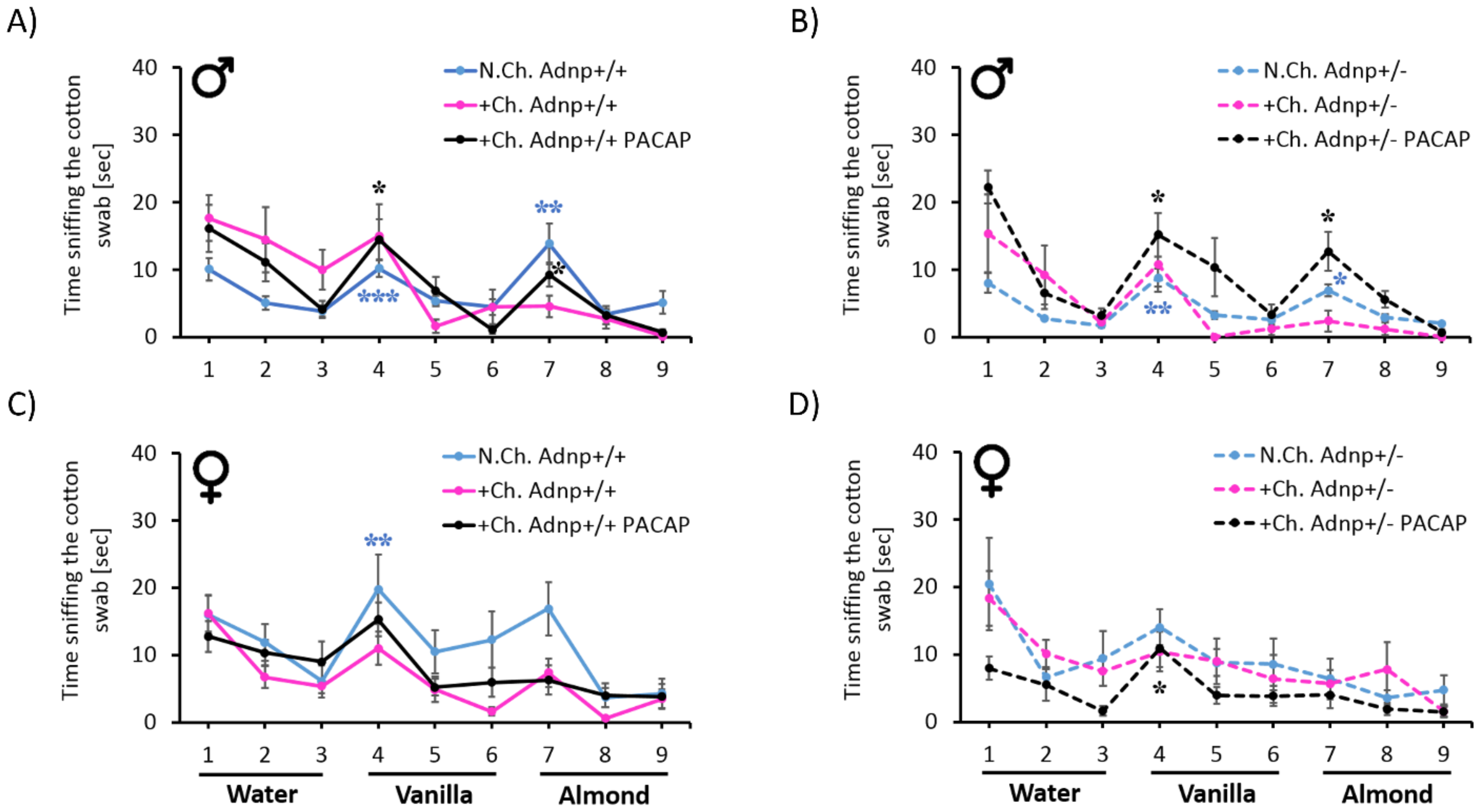

Fig. S1. PACAP restores impaired olfaction in stress-challenged mice.
Paired t-test was performed (males (M): N.Ch. *Adnp*$^{+/+}$ N=18-19; N.Ch. *Adnp*$^{+/-}$ N=12-14; +Ch. *Adnp*$^{+/+}$ N=6-7; +Ch. *Adnp*$^{+/-}$ N=3-4; +Ch. *Adnp*$^{+/+}$ PACAP N=8-9; +Ch. *Adnp*$^{+/-}$ PACAP N=3-4; females (F): N.Ch. *Adnp*$^{+/+}$ N=7; N.Ch. *Adnp*$^{+/-}$ N=5-6; +Ch. *Adnp*$^{+/+}$ N=8-9; +Ch. *Adnp*$^{+/-}$ N=6-7; +Ch. *Adnp*$^{+/+}$ PACAP N=8; +Ch. *Adnp*$^{+/-}$ PACAP N=6-7). **(A-D)** *p<0.05, **p<0.01, ***p<0.001 vs. previous sniffing (novel vs. familiar odor).

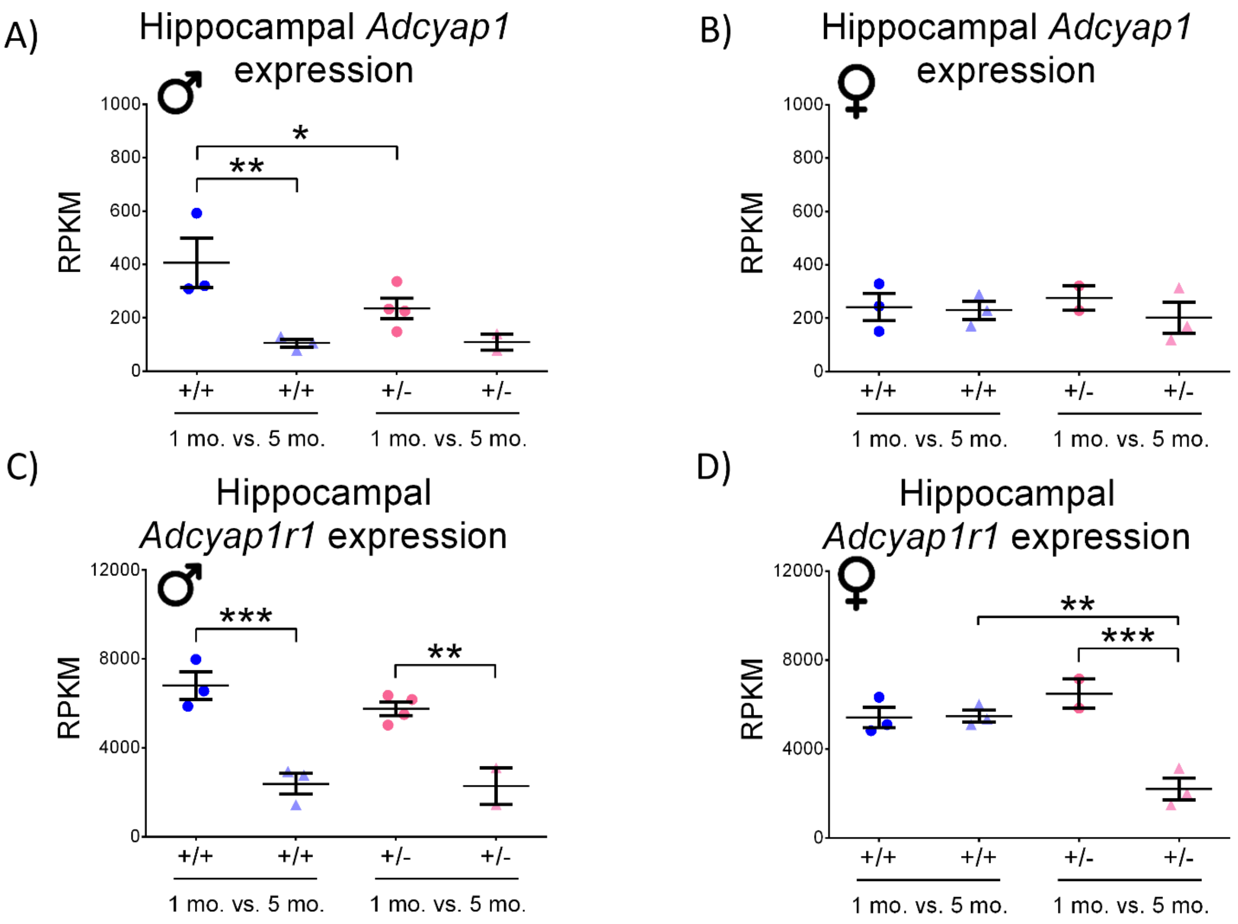

Fig. S2. Hippocampal PACAP (*Adcyap*) and PAC1 receptor (*Adcyap1r1*) gene expression are regulated in age- and genotype-dependent manner in the *Adnp*-haploinsufficient mouse model.

Two-way ANOVA with Tukey post hoc test was performed (males (M): 1-month-old *Adnp*$^{+/+}$ N=3; 1-month-old *Adnp*$^{+/-}$ N=4; 5-month-old *Adnp*$^{+/+}$ N=3; 5-month-old *Adnp*$^{+/-}$ N=2; females (F): 1-month-old *Adnp*$^{+/+}$ N=3; 1-month-old *Adnp*$^{+/-}$ N=2; 5-month-old *Adnp*$^{+/+}$ N=3; 5-month-old *Adnp*$^{+/-}$ N=3). **(A)** Hippocampal *Adcyap* expression in males. A significant main age effect was found (F(1,8)=14.303, p=0.005), with significant differences between 1-month- and 5-month-old *Adnp*$^{+/+}$ mice (**p<0.01), as well as in 1-month-old *Adnp*$^{+/+}$ vs. *Adnp*$^{+/-}$ mice (*p<0.05). **(B)** No significant changes were observed in female hippocampal *Adcyap* gene expression. **(C)** Hippocampal *Adcyap1r1* expression in males. A significant main age effect was found (F(1,8)=57.325, p<0.001), with significant differences between 1-month- and 5-month-old *Adnp*$^{+/+}$ mice (***p<0.001), and *Adnp*$^{+/-}$ mice (**p<0.01). **(D)** Hippocampal *Adcyap1r1* expression in females. Significant main genotype (F(1,7)=5.654, p=0.049), age (F(1,7)=20.854, p=0.003) and interaction (F(1,7)=22.131, p=0.002) effects were found, with significant differences between 1-month- and 5-month-old *Adnp*$^{+/-}$ mice (***p<0.001), as well as in 5-month-old *Adnp*$^{+/+}$ vs. *Adnp*$^{+/-}$ mice (**p<0.01).

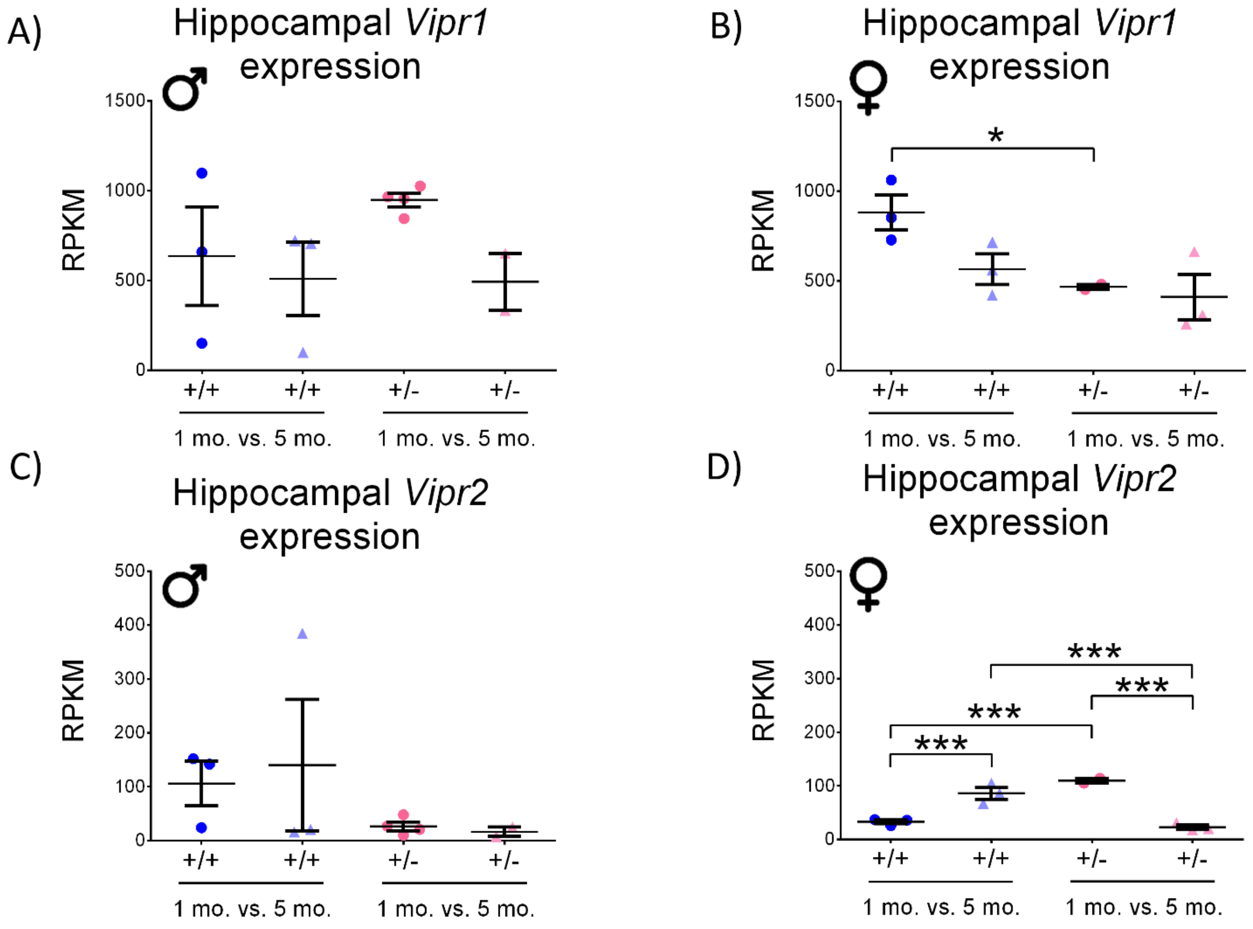

Fig. S3. Hippocampal VIP1 (*Vipr1)* and VIP2 (*Vipr2*) receptor gene expression are regulated in age- and genotype-dependent manner in the *Adnp*-haploinsufficient mouse model.

Two-way ANOVA with Tukey post hoc test was performed (males (M): 1-month-old *Adnp*$^{+/+}$ N=3; 1-month-old *Adnp*$^{+/-}$ N=4; 5-month-old *Adnp*$^{+/+}$ N=3; 5-month-old *Adnp*$^{+/-}$ N=2; females (F): 1-month-old *Adnp*$^{+/+}$ N=3; 1-month-old *Adnp*$^{+/-}$ N=2; 5-month-old *Adnp*$^{+/+}$ N=3; 5-month-old *Adnp*$^{+/-}$ N=3). **(A)** No significant changes were observed in male hippocampal *Vipr1* gene expression. **(B)** Hippocampal *Vipr1* expression in females. A significant main genotype effect was found ($F(1,7)=7.668$, $p=0.028$), with significant differences in 1-month-old *Adnp*$^{+/+}$ vs. *Adnp*$^{+/-}$ mice (*$p<0.05$). **(C)** No significant changes were observed in male hippocampal *Vipr2* gene expression. **(D)** Hippocampal *Vipr2* expression in females. A significant main interaction effect was found ($F(1,7)=97.180$, $p<0.001$), with significant differences between 1-month- and 5-month-old mice of both genotypes (***$p<0.001$), as well as between *Adnp*$^{+/+}$ and *Adnp*$^{+/-}$ mice of both ages (***$p<0.001$).

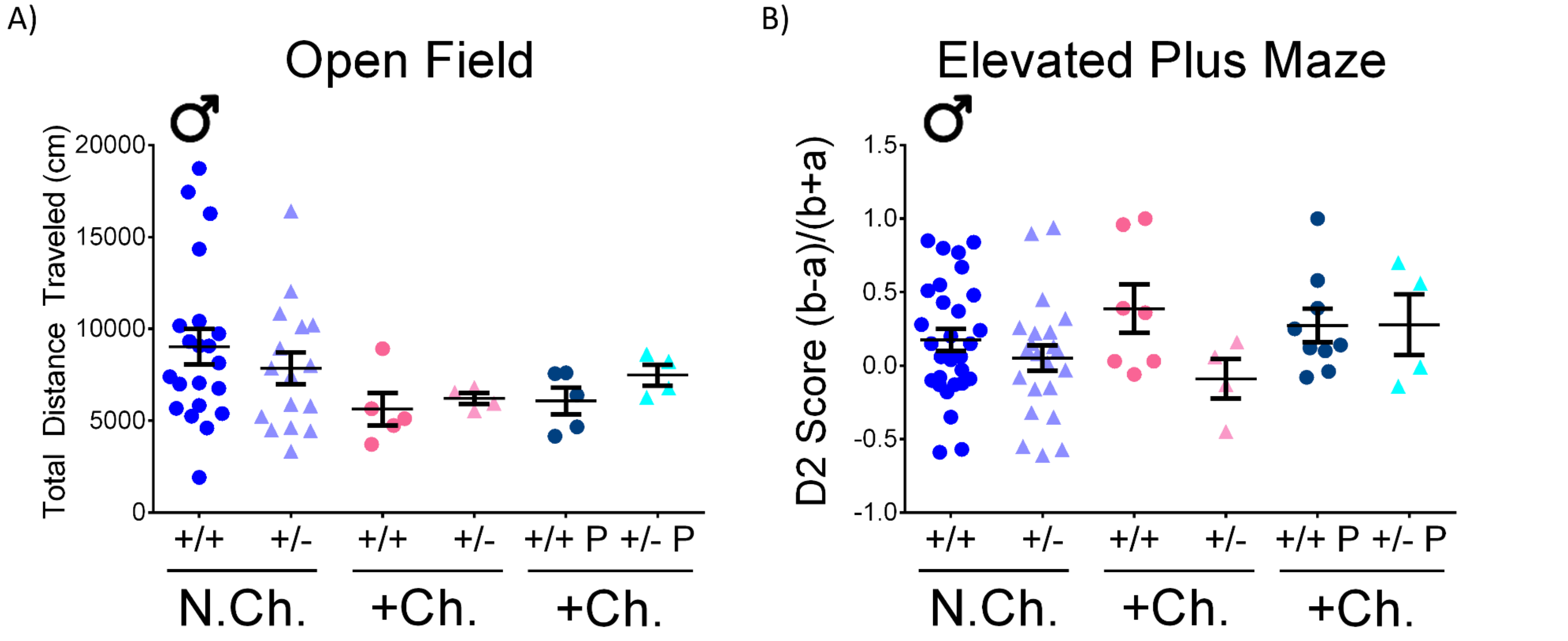

Fig. S4. Male mice do not exhibit any significant effects due to the *Adnp* genotype or PACAP treatment in neither open field nor EPM behavioral tests.

Two-way ANOVA with Tukey post hoc test was performed (males (M): N.Ch. *Adnp*$^{+/+}$ N=21-27; N.Ch. *Adnp*$^{+/-}$ N=16-22; +Ch. *Adnp*$^{+/+}$ N=5-7; +Ch. *Adnp*$^{+/-}$ N=4; +Ch. *Adnp*$^{+/+}$ PACAP N=5-9; +Ch. *Adnp*$^{+/-}$ PACAP N=4). **(A-B)** In males, no significant behavioral alterations were observed in the open field and elevated plus maze tests, either due to stress-challenge or PACAP treatment.

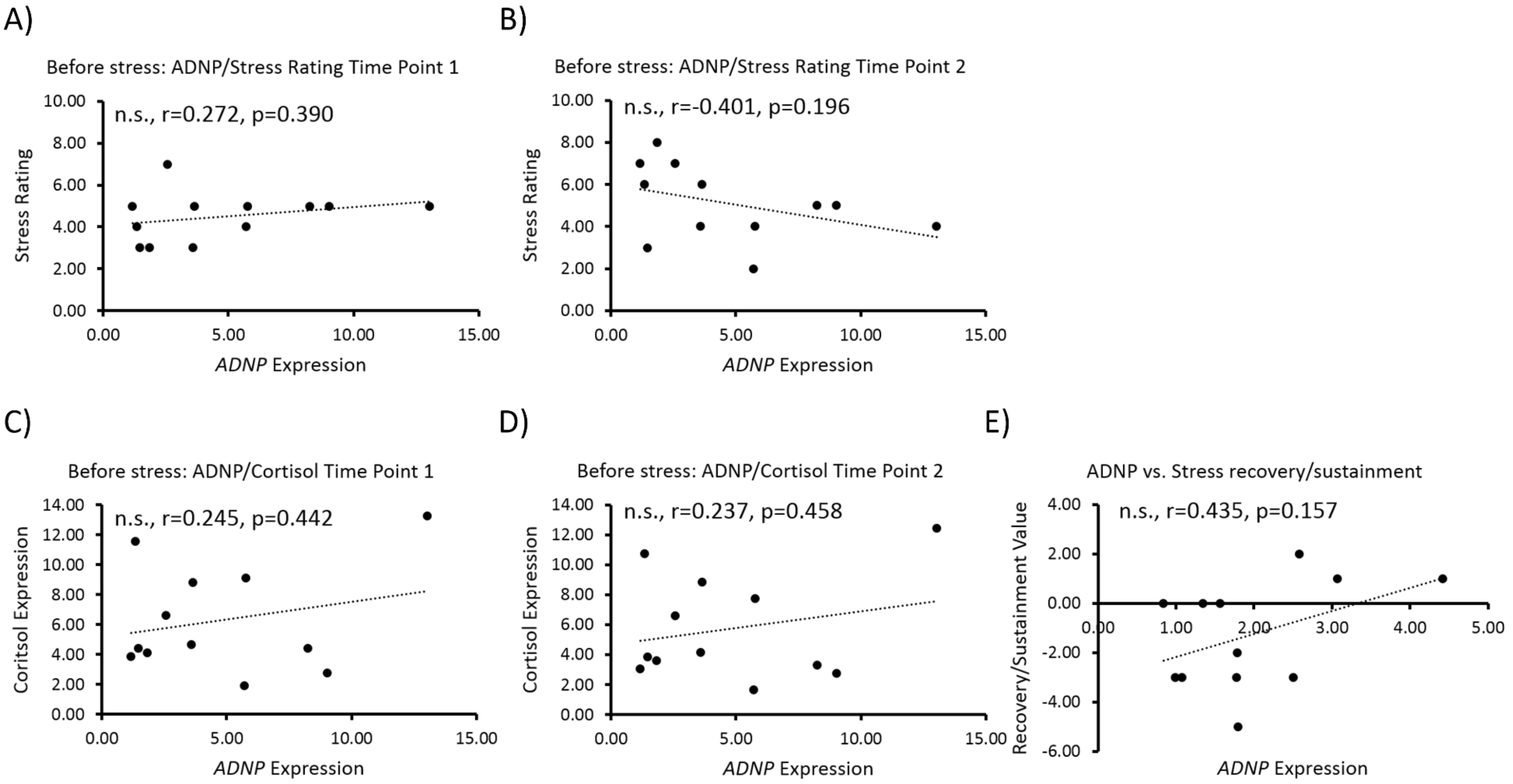

Fig. S5. Human *ADNP* expression is not correlated with stress/salivary cortisol levels before exposure to stress.
In a human cohort (n=12 subjects), data sets of either stress rating (on a 9-point Likert scale) or salivary cortisol levels were plotted against lymphocytic *ADNP* gene expression level. In case both data sets were normally distributed, data were checked for Pearson's correlation. If at least one of the data sets was not normally distributed, data were checked for Spearman's correlation. **(A-D)** Lymphocytic *ADNP* gene expression was not correlated with stress rating/salivary cortisol levels before exposure to stress. **(E)** Although insignificant, lymphocytic ADNP gene expression presented a trend of positive correlation with recovery/sustainment values.

Supplemental Tables:

Table S1: Experimental group allocation (N = Number of animals per experimental group).

Experimental method / Behavioral test	N.Ch. *Adnp*$^{+/+}$		N.Ch. *Adnp*$^{+/-}$		+Ch. *Adnp*$^{+/+}$		+Ch. *Adnp*$^{+/-}$		+Ch. *Adnp*$^{+/+}$ PACAP		+Ch. *Adnp*$^{+/-}$ PACAP	
	**Males**	**Females**	**Males**	**Females**	**Males**	**Females**	**Males**	**Females**	**Males**	**Females**	**Males**	**Females**
Open Field	21	5	16	4	5	6	4	4	5	5	4	4
Elevated plus maze	29	7	22	6	7	9	4	7	9	8	4	7
Object recognition	29	7	24	6	7	9	4	7	9	8	4	7
Social approach task	29	7	21	6	7	9	4	7	8	8	4	7
Odor discrimination	17-19	7	12-14	5-6	6-7	8-9	3-4	6-7	8-9	8	3-4	6-7

Supplemental Materials and Methods:

Peptide synthesis and PACAP treatment: Prior to beginning behavioral tests, intranasal treatment was administered daily over a 1-month period to 9-12-month-old male and female mice (5µg/5µl/mouse/dose). For intranasal administration, the peptide was dissolved in a vehicle solution[1], termed DD, in which each milliliter included 7.5 mg of NaCl, 1.7 mg of citric acid monohydrate, 3 mg of disodium phosphate dihydrate, and 0.2 mg of benzalkonium chloride solution (50%). PACAP or vehicle solution (DD) were administered to mice hand-held in a semisupine position with nostrils facing the investigator. A pipette tip was used to administer 5µl/mouse/dose. The mouse was handheld until the solution was totally absorbed (~10 s). Nasal PACAP application was performed daily, twice a day, 5 days a week. In days of scheduled behavioral tests, PACAP was applied once a day, 2 h before the test.

Open Field: The open field apparatus is a 50×50 cm square arena, with 30 cm high walls, (white/black colored). Mice were individually placed in the open field and left to explore it freely for 15 min. The distance moved and time spent in the entire open field, as well as in its inner defined quadrants (center, border) were recorded using the EthoVision XT video tracking system and software (Noldus Inc. Leesburg, VA, USA).

Elevated Plus Maze (EPM): The elevated plus maze trial is used for testing anxiety, based on the assumption that animals suffer from fear of open spaces. The maze consists of two open arms and two closed arms (50 cm×10 cm×40 cm each). The arms of each type are opposite to each other. The maze is elevated to 50 cm height from the floor. The experiment was conducted in a testing room with a dim light. Mice were placed onto the center of the maze, facing an open arm, and left free to explore it for 5 min. The time spent in the open and closed arms was recorded and compared. The data were analyzed using the following formula: D2=(b-a)/(b+a), in which 'a' designated the time spent in the open arms and 'b' designated the time spent in the closed arms[1]. The longer stay in the closed arms reflects increased anxiety-like behavior[2, 3]. Animals were tracked, monitored and recorded using the EthoVision XT video tracking system (Noldus Inc. Leesburg, VA, USA).

Object recognition: The test included two consecutive days of habituation (5 min per day) and the experimental day which consisted of the three phases. In phase 1 (habituation), the open field

apparatus (50 × 50 cm) contained two identical objects (plastic or metal, 4 × 5 cm^2) and a mouse was placed in the apparatus facing the wall and allowed to freely explore the objects (5 min). After 3 h in the home cage, the mouse was placed back into the apparatus for 3 min for phase 2 (short retention choice phase), during which one of the familiar objects was replaced with a novel object. Approximately 24 h after the completion of phase 2, the mouse was placed into the apparatus for 3 min for phase 3 (long retention choice phase), during which one of the familiar objects was replaced with a novel object. The mouse was kept in its home cage between phases 2 and 3. The time spent sniffing/touching each object was measured. Mouse movement and exploratory behavior were tracked and recorded using the EthoVision XT video tracking system and software (Noldus Inc. Leesburg, VA, USA). Data were analyzed using the discrimination capacity formula: D2 = (b-a)/(a+b), where 'a' designated the time of exploration of the familiar object and 'b' designated the time of exploration of the novel object.

Social approach: Mice used as novel (target mice) to be explored by the subject mice were from the C57BL/6 strain (in our colony), known for their docile nature (6-month-old). The social approach task was previously reported[4-6]. A plexiglas box was divided into three adjacent chambers, each 20 cm (length) × 40.5 cm (width) × 22 cm (height), separated by two removable doors. Steel wire pencil cups (10.16 cm (diameter), 10.8 cm (height)), www.kitchen-plus.com, were used as both containment for the target mice and as inanimate objects (weights prevented the mice from overturning the cups). Experiments were conducted in a dimly lit area during the light phase of the mouse. Target mice (males for males and females for females) were placed inside the wire cup in one of the side chambers for three 10-min sessions on the day before the test for habituation. The next day, each subject mouse was tested in an experiment with three phases, each 10-min long (measured with a timer): I and II, the habituation phases (ensuring no bias), and III, the experimental phase. No significant differences were noted between time periods spent in the different chambers in the habituation phase. In phase III, an empty wire cup (novel object) was placed in the center of the right or left chamber and the cup containing the target mouse was placed in the center of the other chamber. Location of the empty wire cup (novel object) and the novel mice were counterbalanced to avoid confounding side preference. The doors were then removed and a 10-min timer was initiated. The three-chamber apparatus was cleaned between mice. The social approach task was also used as habituation for the social memory task: 3 h after the first

phase (3-min exposure), the mouse was placed back into the apparatus for another 3 min (second phase), during which one cup contained the familiar mouse and the other contained a novel mouse. The positions of the familiar and novel mouse during phases 1 and 2 were counterbalanced within and between groups to exclude the possibility of positional effects but were kept the same for a given animal. Mouse movement and exploratory behavior were tracked and recorded using the EthoVision XT video tracking system and software (Noldus Inc. Leesburg, VA, USA). The discrimination capacity (social memory) was analyzed using the formula: D2 = (b-a)/(b+a).

Odor discrimination: Odors were presented on a suspended cotton swab to the test mouse placed into the clean cage with fresh shavings. Each mouse was tested during three consecutive 2-min periods for each odor, with 2-min intervals between presentations. The x axis indicates the consecutive number of the odor exposure period. The time that the mouse smelled the swab was recorded (beginning whenever the animal oriented its nostrils toward the cotton swab, within 2 cm or less).

Gene expression analysis: 9-12-month-old mouse splenic RNA was extracted using TRI Reagent® (T9424, Sigma-Aldrich, MO, USA). 1μg RNA/sample was then subjected to reverse transcription (RT) using qScript cDNA Synthesis Kit (Quanta Biosciences, Gaithersburg, MD, USA). Further Real-time PCR analysis was performed using PerfeCtaTM SYBR® Green FastMixTM, Low ROXTM (Cat. No. 95074-012, Quanta Biosciences, Gaithersburg, MD, USA) and the QuantStudio 12K Flex Real-Time PCR System (Thermo Fisher Scientific, Waltham, MA, USA). RNA expression levels were determined using specific mouse primers: *Adnp* gene, sense 5′-ACGAAAAATCAGGACTATCGG-3′, anti-sense 5′-GGACATTCCGGAAATGACT-3′. Hypoxanthine-guanine phosphoribosyltransferase (*Hprt*) was selected as a stable reference gene with appropriate primers for mouse, sense 5′-GGATTTGAATCACGTTTGTGTC-3′, anti-sense 5′-AACTTGCGCTCATCTTAGGC-3′. Results are presented as $2^{-\Delta CT}$ [7]. For human analysis, lymphocytic RNA was extracted as previously described[8]. RNA expression levels were determined using specific human primers: *Adnp* gene, sense 5′-ACTTACGAAAAACCAGGACTATC-3′, anti-sense 5′- GACATTGCGGAAATGACT-3′. *Gusb* was selected as a reference gene with appropriate human primers, sense 5′-CTGCTGGCTACTACTTGAAGATG-3′, anti-sense 5′- GAGTTGCTCACAAAGGTCAC-3′.

References:

1. Alcalay RN, Giladi E, Pick CG, Gozes I. Intranasal administration of NAP, a neuroprotective peptide, decreases anxiety-like behavior in aging mice in the elevated plus maze. *Neuroscience letters* 2004; **361**(1-3)**:** 128-131.

2. Pellow S, Chopin P, File SE, Briley M. Validation of open:closed arm entries in an elevated plus-maze as a measure of anxiety in the rat. *Journal of neuroscience methods* 1985; **14**(3)**:** 149-167.

3. Treit D, Menard J, Royan C. Anxiogenic stimuli in the elevated plus-maze. *Pharmacology, biochemistry, and behavior* 1993; **44**(2)**:** 463-469.

4. Malishkevich A, Amram N, Hacohen-Kleiman G, Magen I, Giladi E, Gozes I. Activity-dependent neuroprotective protein (ADNP) exhibits striking sexual dichotomy impacting on autistic and Alzheimer's pathologies. *Transl Psychiatry* 2015; **5:** e501.

5. Amram N, Hacohen-Kleiman G, Sragovich S, Malishkevich A, Katz J, Touloumi O, *et al*. Sexual divergence in microtubule function: the novel intranasal microtubule targeting SKIP normalizes axonal transport and enhances memory. *Molecular psychiatry* 2016; **21**(10)**:** 1467-1476.

6. Hacohen-Kleiman G, Sragovich S, Karmon G, Gao AYL, Grigg I, Pasmanik-Chor M, *et al*. Activity-dependent neuroprotective protein deficiency models synaptic and developmental phenotypes of autism-like syndrome. *J Clin Invest* 2018.

7. Schmittgen TD, Livak KJ. Analyzing real-time PCR data by the comparative C(T) method. *Nat Protoc* 2008; **3**(6)**:** 1101-1108.

8. Vaisvaser S, Modai S, Farberov L, Lin T, Sharon H, Gilam A, *et al*. Neuro-Epigenetic Indications of Acute Stress Response in Humans: The Case of MicroRNA-29c. *PLoS One* 2016; **11**(1)**:** e0146236.
